# Joint Expedition: Exploring the Intersection of Digital Health and AI in Precision Medicine with Team Integration

**DOI:** 10.3390/jpm14040388

**Published:** 2024-04-04

**Authors:** Daniele Giansanti

**Affiliations:** Centro Nazionale Tecnologie Innovative in Sanità Pubblica, Istituto Superiore di Sanità, Via Regina Elena 299, 00161 Roma, Italy; daniele.giansanti@iss.it

## 1. The Joint Expedition Exploring the Intersection of Digital Health and AI in Precision Medicine

Precision medicine stands as a transformative force in the orbit of healthcare, fundamentally reshaping traditional approaches by customizing therapeutic interventions to align with the distinctive attributes of individual patients [1,2]. This revolutionary paradigmatic shift has been propelled forward by the convergence of two pivotal technological frontiers: digital health technologies and the remarkable progress made in artificial intelligence (AI). Together, they forge an unparalleled opportunity to not only refine but also amplify the precision, efficiency, and widespread availability of personalized healthcare. The integration of digital health technologies has played a pivotal role in augmenting the landscape of precision medicine [2,3]. Through the utilization of electronic health records, wearable devices, and other interconnected health monitoring tools, a wealth of patient data has become readily accessible [4]. This important collection of information encompasses genetic profiles, lifestyle choices, environmental factors, and real-time physiological metrics, thereby providing a comprehensive understanding of an individual’s health status. The synthesis of these multifaceted, polyhedric, and complex datasets empowers healthcare practitioners with an all-inclusive perspective, enabling them to craft treatment strategies that are finely tuned to the intricate gradations of each patient’s unique health profile [5,6]. In tandem with the rise of digital health, AI has emerged as a linchpin in the evolution of precision medicine [7,8]. Machine learning algorithms, powered by vast datasets, can discern intricate patterns and correlations within a diverse range of health information. This analytical prowess facilitates the identification of subtle biomarkers, predictive indicators, and personalized treatment responses that might elude traditional diagnostic methods. By harnessing the predictive capabilities of AI, healthcare professionals can not only refine diagnosis and prognosis but also anticipate potential therapeutic outcomes, fostering a more proactive and preemptive approach to patient care.

The integration of digital health and AI technologies in healthcare enhances efficiency by automating routine tasks, streamlining diagnostics, and allowing more time for nuanced patient care [4,7,8]. The rapid assimilation of vast datasets facilitates quicker, more accurate decision-making, reducing the risk of adverse outcomes. This synergy also democratizes personalized healthcare, extending precision medicine beyond specialized centers through the accessibility of digital tools and scalable AI solutions. Remote patient monitoring, telemedicine, and AI diagnostics overcome geographical barriers, improving healthcare inclusivity and addressing disparities. This convergence marks an era of unprecedented healthcare advancements, refining personalized treatments and extending their global reach, with a vision of placing everyone’s unique health profile at the forefront of therapeutic strategies. The initiative to establish a dual opportunity for scientific and editorial collaboration has been initiated with this project’s Special Issue (SI), “Transforming Precision Medicine: The Intersection of Digital Health and AI”. (available at https://www.mdpi.com/journal/jpm/special_issues/4FMFQUN50A, access on 10 March 2024).

This endeavor aimed to create a platform that not only facilitates scientific exchange but also provides a space for editorial collaboration, fostering a comprehensive environment for both scholarly and publishing pursuits.

This Special Issue has successfully achieved a significant milestone, featuring 20 contributions (Co)s (excluding this editorial) [9,10,11,12,13,14,15,16,17,18,19,20,21,22,23,24,25,26,27,28].

**Figure 1:**
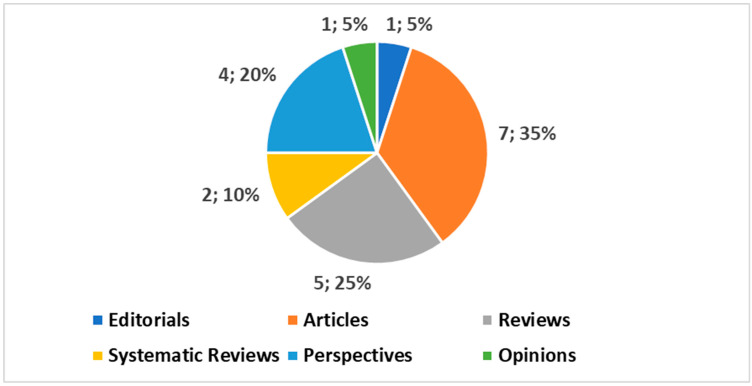
Categories of papers published in this Special Issue.

The published papers (see Figure 1), according to the selected categories, encompass 1 introductory editorial [9], 7 full scientific articles [10,11,12,13,14,15,16], 5 reviews [17,18,19,20,21], 2 systematic reviews [22,23], 4 perspectives [24,25,26,27], and 1 opinion article [28].

## 2. Conclusive Discoveries: A Closer Look at the Contributions

### 2.1. An Overview of the Contributions

Below, we present a concise overview encapsulating the key points and insights from the contributions featured in this Special Issue. This conclusive list aims to provide a brief yet comprehensive glimpse into the diverse and impactful content published within this specialized collection.

The Editorial by Giansanti [9] introduces the aims of the SI and reflects on the progress and status of the introduction of AI into precision medicine. The focus is on assessing the current state and briefly exploring both its evolution and recent trends. The editorial introduces the need for this initiative as a Special Issue that suggests fields and directions for exploration.

Leung et al. [10] explore the use of planning computed tomography (pCT)-based radiomics for the long-term prognostication of high-risk localized prostate cancer patients who underwent whole pelvic radiotherapy (WPRT). Given the high mortality rate of high-risk prostate cancer and challenges with traditional prognostic markers, the research employed rigorous radiomics methodologies on a cohort of 64 patients. The pCT-based radiomics model demonstrated a consistent and comparable performance to MRI-based studies, predicting six-year progression-free survival with a mean AUC of 0.76 (training) and 0.71 (testing). The radiomics signature, incorporating two texture features, exhibited promising accuracy, sensitivity, and specificity in both training and testing cohorts. This study suggests that pCT-based radiomics could serve as a routine, non-invasive approach for prognostic prediction in high-risk localized prostate cancer cases undergoing WPRT, leveraging the accessibility of CT in standard clinical practices.

The retrospective study proposed by Morena et al. [11] utilizing artificial intelligence aimed to assess the impact of the COVID-19 pandemic on pulmonary tuberculosis (TB). Analyzing electronic health records from Spain’s Castilla-La Mancha region, this study compared data from 2015 to 2020. In 2020, pulmonary TB diagnoses decreased by 28% compared to 2019, with 14.2% of patients diagnosed with both TB and COVID-19. Despite a higher risk for coinfection among women, symptoms were no more severe than those with isolated TB. The findings suggest a notable decline in pulmonary TB incidence during the initial year of the COVID-19 pandemic.

Issa et al. [12] investigate the impact of cone-beam computed tomography (CBCT) viewing parameters on the identification of the inferior alveolar nerve (IAC). The study assessed 25 CBCT scans, testing different slice thicknesses, sharpness, and contrast settings. A three-score system evaluated IAC visibility. Optimal parameters were determined, and validation was conducted through semi-automated segmentation and structure overlapping, assessing the mean distance. Inter-rater and intra-rater reliability were significant (69–83%). A 0.25 mm slice thickness, zero sharpness, and contrast of 1200 consistently improved the visibility and accuracy. The consideration of individual patient characteristics is recommended when applying these parameters, including anatomical variations and bone density.

Griewing et al. [13] recall how the rising accessibility of large language models (LLMs) has sparked interest in utilizing generative AI applications for medical purposes. Their observational study addresses the use of LLM ChatGPT 3.5 for treatment recommendations in breast cancer, comparing outcomes with a multidisciplinary tumor board (MTB). Incorporating patient profiles that reflected diverse breast cancer stages, including precancerous lesions and metastasis, the study found an overall concordance of 50%, rising to 58.8% for invasive breast cancer profiles. However, due to occasional fraudulent decisions by the LLM, the study concludes that publicly available LLMs are currently insufficient as support tools for tumor boards. Gynecological oncologists are encouraged to familiarize themselves with LLM capabilities, considering potential risks and limitations while exploring their potential utility.

Aiumtrakul et al. [14] explore the use of AI tools such as ChatGPT, Bing Chat, and Bard AI in the literature through searches on nephrology, specifically evaluating their citation accuracy. The researchers generated prompts to obtain references in Vancouver style for 12 nephrology topics from each AI tool and assessed their validity using PubMed, Google Scholar, and Web of Science. The results reveal varying levels of citation accuracy, with ChatGPT providing 38% accurate references, Bing Chat 30%, and Bard AI only 3%. Common errors included incorrect DOIs. This study underscores the importance of research integrity in medicine and emphasizes the need for refined AI tools before their widespread adoption in medical literature searches.

The work by Elvas et al. [15] addresses the significant global burden of cardiovascular diseases (CVDs), specifically focusing on myocardial infarction, pulmonary thromboembolism, and aortic stenosis. Utilizing data from Hospital Santa Maria, their research employs a comprehensive approach integrating exploratory data analysis (EDA) and predictive machine learning (ML) models. Following the Cross-Industry Standard Process for Data Mining (CRISP-DM) methodology, EDA uncovers intricate patterns and relationships specific to cardiovascular diseases. ML models exhibit accuracies exceeding 80%, providing a 13 min window for predicting myocardial ischemia incidents and enabling proactive interventions. This paper establishes a proof of concept for real-time data and predictive capabilities, offering valuable tools for informed decision making and timely interventions in managing cardiovascular diseases.

Pereira et al. [16] recall how chronic obstructive pulmonary disease (COPD) stands as the third leading cause of global mortality, necessitating effective management strategies. Their study highlights the pivotal role of Health Remote Monitoring Systems (HRMSs) in COPD patient care, employing artificial intelligence (AI) models to predict health deterioration risks by analyzing biometric signs and environmental factors. The research not only reviews recent works in this domain but also introduces an Intelligent Clinical Decision Support System (CIDSS). Comprising vital signs of prediction and early warning score calculation modules, the CIDSS generates early information on patient health evolution and risk analysis. It issues alerts for anomalies in biometric measurements or significant basal value changes, enabling proactive intervention. This system was implemented, assessed in a real case, and validated through an evaluation survey by healthcare professionals, affirming its utility and value in facilitating adjustments to COPD patient treatment. The CIDSS emerges as a valuable tool for medical professionals, supporting proactive healthcare interventions.

The review proposed by Iannone and Giansanti [17] explores the integration of artificial intelligence (AI) with assistive technologies (ATs) in the context of autism, recognizing the need for a multidisciplinary approach to diagnosis and therapy. A systematic review of 22 studies revealed promising interest in AI integration, particularly in AI robotics and wearable automated devices like smart glasses. These innovations hold substantial potential for enhancing communication and social engagement among individuals with autism. However, the emphasis on innovation over establishing a solid presence in healthcare raises concerns about regulatory and acceptance issues. As the field evolves, it becomes evident that integrated ATs with AI play a pivotal role in connecting various domains and addressing the complexities of autism.

Miao et al.’s review [18] discusses the significant impact of artificial intelligence (AI), particularly machine learning, on nephrology and the management of kidney diseases. It specifically focuses on ChatGPT, an innovative language model developed by OpenAI. The article highlights ChatGPT’s versatility in engaging in informative conversations and its demonstrated proficiency in medical knowledge assessments. While acknowledging its varying performance across medical subfields, this review provides an overview of ChatGPT’s integration in nephrology, exploring its potential benefits in dataset management, diagnostics, treatment planning, patient communication, and medical research. Ethical and legal concerns are discussed, emphasizing the importance of thorough evaluation before implementing AI in real-world medical scenarios. This review aims to be a valuable resource for nephrologists and healthcare professionals interested in utilizing AI for personalized nephrology care.

Ittarat et al. [19] highlight in their review how the integration of ophthalmology chatbots in modern eye care represents a significant technological advancement, offering benefits such as improved access to information, enhanced patient interaction, and streamlined triaging. Evaluations have demonstrated their effectiveness in ophthalmology condition triage and knowledge assessment, highlighting both their potential and areas for improvement. Challenges in integrating these chatbots into healthcare systems include ethical, legal, and integration issues. Future developments, including the synergy of artificial intelligence and machine learning, promise to enhance their diagnostic capabilities globally. This *review* explores the utilization of ophthalmology chatbots, assessing their accuracy, reliability, data protection, security, transparency, potential biases, and ethical considerations. It provides a comprehensive review of their roles in ophthalmology, emphasizing their significance and future potential in the field.

Fawaz et al. [20] propose a review that recalls how cancer is a leading cause of global disease-related death, emphasizing the importance of accurate early diagnosis and intervention. Traditional diagnostic methods include clinical examination, biomarker blood tests, biopsy histopathology, and imaging. The review highlights that the integration of diverse omics data, such as genomic, metabolomic, and microbiomic traits, is challenging and carries a risk of interpretation errors. Systems biology, combining artificial intelligence (AI) with omics technologies, is presented as a solution to analyze and integrate vast patient data, aiding physicians in diagnosis and treatment decisions rapidly and accurately. The article acknowledges the potential of AI in cancer research but highlights the associated risks, including diagnostic and prognostic errors in data interpretation.

Meijer et al.’s review [21] recalls that digital twin technology stands out as a promising advancement in healthcare, offering applications in monitoring, diagnosis, and personalized treatment strategies. It explores digital twins as virtual counterparts of real human patients, aiming to provide an in-depth understanding of the data sources and methodologies contributing to their construction across various healthcare domains. The review covers diverse data sources, such as blood glucose levels, heart MRI and CT scans, cardiac electrophysiology, written reports, and multi-omics data. Each source presents challenges related to standardization, integration, and interpretation, but the review showcases how various datasets and methods are used to overcome these obstacles and generate a digital twin. Despite significant progress, challenges remain in achieving a fully comprehensive patient digital twin. The article discusses critical developments in non-invasive data collection, high-throughput technologies, modeling, and computational power. Overall, while facing challenges, digital twin research holds great promise for personalized patient care and has the potential to shape the future of healthcare innovation.

Allen [22] proposes a systematic review, providing a synthesis of the literature on explaining machine-learning models for digital health data in precision medicine. As healthcare increasingly customizes treatments to individual characteristics, the integration of artificial intelligence with digital health data becomes crucial. Utilizing a topic-modeling approach, this paper distills key themes from 27 journal articles. Topics identified include optimizing patient healthcare through data-driven medicine, predictive modeling with data and algorithms, predicting diseases with the deep learning of biomedical data, and machine learning in medicine. The review explores specific applications of explainable artificial intelligence, emphasizing its role in fostering transparency, accountability, and trust within the healthcare domain. It underscores the need for the further development and validation of explanation methods to advance precision healthcare delivery.

Michelutti et al.’s systematic review [23] aims to analyze the primary reports on the utilization of artificial intelligence algorithms in the medical field, with a specific focus on oncology, particularly in the context of prognostic evaluations for patients with head and neck malignancies. The objective is to comprehensively examine the existing literature pertaining to the application of artificial intelligence in head and neck oncology, specifically for prognostic assessments. The review provides an encompassing overview of how artificial intelligence is employed to derive prognostic information, particularly in predicting survival and recurrence. These findings underscore the potential impact of these prognostic data on tailoring therapeutic strategies to become increasingly personalized.

Lone et al. [24] proposed a perspective focusing on anterior open-bite malocclusion, a dental condition characterized by a lack of contact between the upper and lower front teeth, leading to functional difficulties. Etiology involves genetic, environmental, and developmental factors. Genetic studies have identified genes and pathways related to jaw growth, tooth eruption, and dental occlusion contributing to open-bite development. Orthodontic treatment, including braces and clear aligners, is a primary approach, with adjuvant therapies and, in severe cases, surgical interventions. Technological advancements like 3D printing enhance treatment precision. Genetic research, especially using animal models like the collaborative cross-mouse population, provides insights into the genetic basis of open bite and potential therapeutic targets. Proposing human research using mouse models, including GWAS, EWAS, RNA-seq analysis, and integration of genetic and expression studies, aims to uncover novel genes and factors influencing open bite, paving the way for more precise treatments and preventive strategies.

Fuchs et al.’s perspective article [25] provides an in-depth exploration of the transformative era unfolding in sarcoma care, propelled by the intersection of digital health and artificial intelligence (AI). It examines the multifaceted opportunities and challenges associated with harnessing these technologies for precision and value-based sarcoma care. The article outlines the current state-of-the-art methodologies and technologies in sarcoma care, offering practical insights for healthcare providers, administrators, and policymakers. Emphasis is placed on the limitations of AI and digital health platforms, underscoring the crucial need for high-quality data and ethical considerations.

Watted et al. [26] present a perspective that delves into the malocclusion phenotype known as deep bite, characterized by the excessive overlap of the upper front teeth over the lower front teeth. It discusses current clinical treatment strategies, explores genetic analyses related to the phenotype, and proposes a roadmap for future genetic investigations. The research underscores the potential of understanding genetic and epigenetic factors for developing new preventive and treatment methods, incorporating technological advancements like 3D printing and CAD/CAM. The study suggests conducting comprehensive genomic analyses, including GWAS and RNAseq, in human tissues associated with deep-bite malocclusion. The collaborative cross-mouse model is highlighted as a valuable tool for identifying genetic factors, paving the way for personalized medicine and early prevention strategies.

The study by Lone et al. [27] explores malocclusion, a prevalent condition influenced by genetic, environmental, and oral behavioral factors, impacting oral functionality, aesthetics, and quality of life. Recognizing the significance of managing malocclusion in primary dentition, this review highlights its global prevalence and the use of Angle’s classification system. Genetic factors, including variants in genes like MSX1, PAX9, and AXIN2, are associated with an increased risk of Class I occlusion. The review aims to provide insights into clinical strategies, genetic influences from human and murine populations, and RNA alterations in skeletal Class I occlusion. Mouse models are crucial for investigating genetic associations and mandible development.

Wiedermann et al. [28] present an opinion delving into the role of artificial intelligence-driven symptom checkers in addressing the challenges faced by modern healthcare, particularly in the context of an aging population and a decreasing general practitioner workforce. Drawing insights from a study in Italian general practices, the article explores the perspectives of both physicians and patients regarding the efficiency, utility, and challenges of symptom checkers. While these tools are seen as potential solutions, concerns about accuracy and misdiagnosis persist. The article proposes that AI-based symptom checkers can optimize medical history-taking, emphasizing the need for the careful integration of digital innovations while preserving the essential human touch in healthcare. Collaboration among technologists, clinicians, and patients is crucial for the successful evolution of digital tools in healthcare.

### 2.2. Common Message

All these works have made notable contributions to the field of personalized medicine, particularly at the intersection between AI and digital health. These contributions provide valuable insights and innovative approaches, contributing to our understanding of how AI and digital health can enhance personalized medicine. The integration of the technologies showcased in these studies offers practical implications for patient care, treatment strategies, and medical decision making, contributing to the ongoing progress in this field.

Twenty distinct contributions [9,10,11,12,13,14,15,16,17,18,19,20,21,22,23,24,25,26,27,28] weave through the intricate fields of the health domain focused on the integration of digital health and AI with precision medicine. These studies, spanning diverse medical domains, collectively leverage several AI and digital health approaches to precision medicine.

The scientific articles [10,11,12,13,14,15,16] offer a glimpse into the current priorities of scholars, with a distinct focus on the integration of digital health and AI within the realm of precision medicine. These articles illuminate the ongoing efforts of researchers, underscoring the increasing importance of incorporating advanced technologies into healthcare practices.

Transitioning to review studies [17,18,19,20,21], they play a critical role in providing essential scientific insights into the consolidation of themes. Importantly, these reviews highlight the pivotal role of AI in shaping the landscape of precision medicine, emphasizing its significance in guiding the current trajectory of research and knowledge consolidation.

Furthermore, homing in on systematic reviews [22,23], these works systematically identify patterns and scientific questions, offering a focused examination of specific areas where scholars are directing their attention. The thematic emphasis on digital health and AI becomes even more apparent, illustrating their integral role in addressing precise scientific inquiries.

Shifting to perspectives [24,25,26,27] and opinions [28], these contributions offer forward-looking insights from various angles, accentuating the dynamic landscape of digital health and AI in precision medicine. By opening up future possibilities, they significantly contribute to our understanding of potential directions and opportunities in this rapidly evolving field.

*In essence*, this collection not only paints a comprehensive picture of the current scholarly focus [10,11,12,13,14,15,16,17,18,19,20,21,22,23,24,25,26,27,28] but also underscores the central role of digital health and AI in advancing precision medicine. The thematic emphasis on these key elements reflects both the present intellectual climate [10,11,12,13,14,15,16,17,18,19,20,21,22,23] and the anticipated trajectories of research in this dynamic domain [24,25,26,27,28].

In this comprehensive exploration of AI in precision medicine, each contribution [9,10,11,12,13,14,15,16,17,18,19,20,21,22,23,24,25,26,27,28] unfolds a distinct facet of the evolving landscape. The focus extends from specialized areas such as radiomics [10] and the TB impact assessment [11] to the meticulous consideration of CBCT parameters [12] and the potential but cautious integration of large language models (LLMs) in breast cancer treatment decisions [13].

The significance of AI tools surfaces in nephrology searches in the literature [14], while the real-time predictive capabilities for cardiovascular diseases (CVDs) [15] promise proactive interventions. Chronic obstructive pulmonary disease (COPD) management [16] and AI’s role in autism treatment [17] underscore the transformative impact of healthcare.

The panorama broadens as nephrology’s interaction with AI [18] and the integration of ophthalmology chatbots [19] reveal a dynamic diagnostic landscape. AI’s potential in cancer research [20] and the promising applications of digital twins in healthcare [21] reflect ongoing advancements and the need for careful ethical considerations.

The narrative unfolds further as we delve into AI’s explainable role [22] and the prognostic applications in oncology [23], highlighting transparency and personalized patient care. Dental perspectives offer insights into malocclusions [24,26,27], while sarcoma care’s transformation [25] and the role of AI-driven symptom checkers [28] provide a conclusive glance at the future.

This Special Issue not only encapsulates the current state of AI in precision medicine but also lays the foundation for an exciting and dynamic future, emphasizing collaborative efforts between technology, healthcare professionals, and patients.

### 2.3. Key Emerging Themes and Suggestions for a Broader Investigation

From the overview, it is also possible to detect the emerging themes (Table 1) and the suggestions for a broader investigation.

The studies also reveal intriguing scientific insights for future research initiatives and expansions. In the exploration of the transformative landscape of precision medicine, interdisciplinary collaborations emerge as a pivotal theme [10,11,12,13,14,15,16,17,18,19,20,21,22,23,24,25,26,27,28]. Opportunities abound for AI experts, healthcare professionals, geneticists, and data scientists to forge synergies, fostering a holistic approach to personalized healthcare.

Ethical considerations take a central role in this evolving paradigm [22]. Delving into issues of patient privacy, data security, and equitable access, a thorough examination ensures the responsible integration of AI and digital health in precision medicine. Patient-centricity takes the spotlight [24,27], urging exploration into tailoring precision medicine to individual needs. Active patient involvement in decision making becomes a crucial aspect, aligning treatments with personal preferences and values. A global perspective unfolds [23,26], revealing diverse adoption patterns of precision medicine practices worldwide. Comparative analyses shed light on the challenges and successes within varying healthcare systems driven by AI approaches to personalized care. The long-term impact of AI and digital health reverberates through the discourse [16,28]. Factors such as cost-effectiveness, scalability, and sustainability are scrutinized, offering insights into strategies to overcome barriers and pave the way for widespread adoption. Integration with public health initiatives is a theme of paramount importance [15,21], outlining the potential role of AI-fueled precision medicine in the early detection, prevention, and management of diseases at the population level. Education and training for healthcare professionals come into focus [17,18], prompting an assessment of the current landscape. Strategies for incorporating relevant skills into medical and allied health curricula are proposed, envisioning a workforce that is prepared for the future of healthcare. Unraveling health disparities is a key exploration [11,20], scrutinizing AI-driven precision medicine’s impact on existing inequalities. Strategies to ensure equitable access and benefits across diverse populations become integral to the evolving narrative. Regulatory frameworks and policies take center stage [13,19], highlighting the governance needed for the ethical and responsible use of AI in precision medicine. Collaborations on an international scale are investigated to establish common guidelines. The dynamic theme of continuous monitoring and feedback emerges [12,14], advocating real-time data integration into precision medicine models. The adaptation and improvement of treatment strategies over time become integral to the ongoing narrative.”

## 3. Conclusions

In conclusion, the evolution of artificial intelligence technologies and digital health in the field of precision medicine offers promising prospects for enhancing patient outcomes and revolutionizing healthcare practices. The studies presented in this editorial highlight the growing intersection between cutting-edge technologies and personalized medicine. The research emphasizes the transformative potential of AI and digital health in driving precision medicine toward unprecedented levels of accuracy and efficiency.

This Special Issue significantly contributes to various domains, identifying both emerging and established themes and delineating intriguing directions for future advancements in digital health and AI in personalized medicine. This initiative underscores the importance of these editorial collections as a central hub for scholarly exchange and discussions among researchers worldwide, fostering collaboration and innovation in the ever-evolving landscape of precision medicine.

## Figures and Tables

**Table 1:** Dominant emerging themes by study.

Themes	Description	Studies	
Cancer Research	Leung et al. [10], Griewing et al. [13], Fuchs et al. [25],	[10,13]	[25]
Pandemic Impact and Disease Dynamics	Morena et al. [11], Issa et al. [12]	[11,12]	
Cardiovascular and Pulmonary Insights	Elvas et al. [15], Pereira et al. [16]	[15,16]	
Neurological Disorders and Autism	Iannone and Giansanti [17]	[17]	
Nephrology and Healthcare Literature	Miao et al. [18], Aiumtrakul et al. [14]	[14,18]	
Ophthalmology Chatbots	Ittarat et al. [19]	[19]	
Cancer Diagnosis and AI Integration	Fawaz et al. [20]	[20]	
Digital Twin Technology in Healthcare	Meijer et al. [21]	[21]	
Dental Research	Lone et al. [24], Watted et al. [26], Lone et al. [27]	[24,26,27]	
Automatic Symptom Checking	Wiedermann et al. [28]	[28]	
Explainable AI in Precision Medicine	Allen [22]	[22]	
Oncology and Prognostic Evaluations	Michelutti et al. [23]	[23]	
